# Antibodies against human influenza viruses in *Galliformes* order

**Published:** 2011-01-10

**Authors:** Behnaz Heydarchi, Masoumeh Tavassoti Kheiri, Seyed Masoud Hosseini, Nariman Sheykhi, Seyedeh Fahime Mousavi, Reza Khaltabadi Farahani, Rouzbeh Bashar

**Affiliations:** 1 Influenza Unit, Pasteur Institute of Iran, Tehran, Iran; 2 Department of Microbiology, Faculty of Biological Sciences, Shahid-Beheshti University, Tehran, Iran; 3 Department of Clinical Sciences, Faculty of Specialized Veterinary Science, Science and Research Campus, Islamic Azad University, Tehran, Iran

## 

Influenza A virus is always concerned as an infectious agent in birds and other mammalian such as humans. Although it is believed that barriers among various species restrict the transmission of influenza viruses, there are evidences implying that origin of all human influenza viruses is derived from avian influenza viruses. To determine the prevalence of human H1 and H3 viruses in the captive birds of *Galliformes* order, a serological surveillance was carried in Tehran Zoo, Saiee Park and Pardisan Park of Tehran, from November 2008 to February 2009. Sera samples were collected from 7 species including Chickens, Guinea fowls, Partridges, Pheasants, Turkeys and Quails and presence of antibodies was detected by haemagglutination inhibition assay. Sera of chickens immunized by human influenza vaccine were used as positive control in the assays. In total, 84.61% and 100% of sera samples had antibodies against human influenza H1 and H3 viruses, respectively. The Geometric Mean Titer (GMT) value for H1 antibodies was 30.33 whereas that related for H3 antibodies was 57.36. Significantly the highest GMT value and the greatest antibody titers were observed in chicken species. As HI assay is able to detect haemagglutination antibodies as soon as 2 weeks to 1 year post-infection, the results of this study indicate that seropositive captive birds were infected during recent year with H1 and H3 virus strain, closely related to human strains. Moreover, as the peak of influenza epidemic in human population in Tehran was at the same time with our sample collection, it could be concluded that influenza viruses’ transmission between human and captive birds occurred.

